# Copper nano-architecture topical cream for the accelerated recovery of burnt skin†

**DOI:** 10.1039/d2na00786j

**Published:** 2023-01-18

**Authors:** Maria Laura Ermini, Maria Summa, Agata Zamborlin, Valentina Frusca, Ana Katrina Mapanao, Enrico Mugnaioli, Rosalia Bertorelli, Valerio Voliani

**Affiliations:** a Center for Nanotechnology Innovation@NEST, Istituto Italiano di Tecnologia Piazza San Silvestro 12 – 56127 Pisa Italy valerio.voliani@unige.it; b Translational Pharmacology, Istituto Italiano di Tecnologia Via Morego 30 – 16163 Genoa Italy rosalia.bertorelli@iit.it; c NEST-Scuola Normale Superiore Piazza San Silvestro 12 – 56127 Pisa Italy; d Department of Earth Sciences, University of Pisa Via S. Maria 53 56126 Pisa Italy; e Department of Pharmacy, University of Genoa Viale Cembrano 4 – 16148 Genoa Italy; f Institute of Life Sciences, Scuola Superiore Sant’Anna Piazza Martiri della Libertà 33 56127 Pisa Italy; g Center for Radiopharmaceutical Sciences, Paul Scherrer Institute 5232 Villigen-PSI Switzerland

## Abstract

Skin burns are debilitating injuries with significant morbidity and mortality associated with infections and sepsis, particularly in immunocompromised patients. In this context, nanotechnology can provide pioneering approaches for the topical treatment of burnt skin. Herein, the significant recovery of radiation-damaged skin by exploiting copper ultrasmall-in-nano architectures (CuNAs) dispersed in a home-made cosmetic cream is described and compared to other noble metals (such as gold). Owing to their peculiar design and components, CuNAs elicit a substantial recovery from burned skin in *in vivo* models after one topical application, and a significant anti-inflammatory effect is highlighted by reducing cytokine expression. The treatment exhibited neither significant toxicity nor the alteration of copper metabolism in the target organs because of the CuNA biocompatibility. This study may open new horizons in the treatment of *radiation dermatitis* and skin burns caused by other external events.

## Abstract 2

This study demonstrates the potential of copper nanotechnology for the topical treatment of burn injuries and opens new horizons in oncological dermatology.

## Graphical abstract

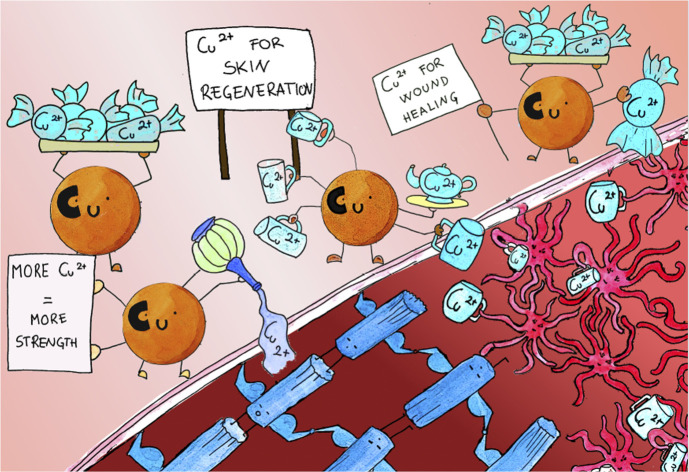

## 1. Introduction

Skin burn injuries represent a global public issue causing 180 000 deaths every year according to the World Health Organization (WHO).^1,2^ Recent epidemiological studies have shown that cases of burns tend to increase globally. Each country suffers from a constant healthcare burden caused by burn cases, particularly the countries with lower sociodemographic index levels are more affected (*i.e.* low per capita income and low average years of education).^3^ Indeed, skin burn can lead to morbidity and mortality from severe infection occurrence. Even if the total number of cases increases globally, the mortality and age-standardized rates of burn incidence continuously decrease owing to the development of specific treatments.^3^

The burn causes an inflammatory state generating histamine release and a consequent fast oedema formation, increased extravascular osmotic activity and vasodilation. The damage to the cells activates the enzyme-mediated hydrolysis of the prostaglandin precursor, leading to the formation of prostaglandin. Consequently, the release of norepinephrine is inhibited by regulating the injury response. Overall, an increase in vacuoles and open endothelial intercellular junctions is observed. Traumatized tissue suffers from a continuous loss of fluids, which often leads to a rapid fall in plasma volume and shock.^4^ Severe complications can arise from the low integrity of the tissue, which is particularly susceptible to the colonization of microbes.^5,6^

The gravity of the injury is categorized based on its etiology and depth. The deep layers are usually protected by the superficial skin, acting as a barrier for reducing heat transfer. However, damage to the underlying tissues can still result in necrosis.^7^

Early treatments may reduce the occurrence of complications. The actual therapeutic strategies, even if sub-optimal include antibacterial drugs, stem cell therapy or application of growth factors, such as transforming growth factor (TGF), fibroblast growth factor (FGF), platelet-derived growth factor (PDGF) and vascular endothelial growth factor (VEGF), to stimulate cell proliferation.^2,8^

In this regard, nanotechnology has been providing incisive advances in skin care, which attracted the interest of a growing number of cosmetic companies. In particular, the design of innovative nanomaterials can result in improved dermal penetration, localized therapeutic action, controlled release of therapeutics, and prolonged residence of the active agents in the skin.^9–13^ For example, gel-based matrixes, sponges and nanofibers have been proposed for promoting the healing process and as antibacterial dressing.^14–16^ The inclusion of metal ions or inorganic nanoparticles has enhanced the benefits of these materials in terms of hemostasis, inflammation and proliferation.^14,17^ Moreover, several inorganic nanoparticles are already employed in cosmetics and skin products, such as titania and zinc oxide nanoparticles in sunscreens, and silica nanoparticles for matte finish makeup.^11,18^ Metal-based nanomaterials are of special interest in cosmeceutics owing to the intrinsic features that can elicit anti-aging and antimicrobial actions.^11^ Among other noble metals, such as gold and silver, copper is particularly attractive because of its peculiar antimicrobial, antifungal, and dermal regeneration properties.^19,20^ Copper stimulates the activity of growth factors and contributes to a series of cascade effects that accelerate the metabolism of the cells and the wound-healing process.^20,21^ In this regard, copper salt medications have demonstrated significant effects in wound healing by stimulating the production of collagen and elastin and inducing the proliferation of fibroblasts.^20,22^ However, treatments based on copper nanomaterials are still in their infancy, and only one product is present in the cosmetic market (a nanoparticle-impregnated pillow that reduces facial wrinkles).^11^

A general distrust arose concerning the employment of noble metal nanomaterials for topical applications because of the lack of information on potential toxicity and biodistribution. Nowadays, the Food and Drug Administration has issued several guidance documents referring to the use of nanotechnology in cosmetics, and the resolution of the European Parliament “*Regulatory aspects of nanomaterials*” states that “*the use of nanomaterials should be guaranteed to the public, while guaranteeing safety*”.^23^ Indeed, neither enough pieces of evidence nor uniform and comprehensive scientific data have been reported yet regarding the potential toxicity and biokinetics of noble metal nanomaterials, and even less on copper for topical applications.^21,24,25^ In this regard, ultrasmall-in-nano architectures (NAs) have been recently described as ideal candidates for the clinical translation of metal nano-therapeutics owing to their intrinsic safety-by-design associated with the efficient clearance or metabolization of the building blocks that avoid the metal persistence in the body.^24,26,27^ NAs enclosing gold have been primarily developed for the non-invasive treatment of oral carcinoma.^28,51^

Herein, we present rationally designed copper-containing nano-architectures (CuNAs) as an active agent of a home-made cosmetic cream for the effective treatment of burnt skin. The rational design of biodegradable NAs allows for good skin penetration together with long-term action.^29^ Copper has been selected as an active agent owing to the intense wound-healing effect associated with the release of ions in the physiological environment.^20^ Both a significant skin regenerative effect and an effective reduction in local inflammation have been confirmed *in vivo* after a single topical application of the CuNA-cream. The biodistribution of copper after treatment has been assessed, underlying a negligible modification of copper metabolism in the target organs. The treatment did not induce substantial toxicity either *in vitro* or *in vivo*. These findings pave the way for establishing a new paradigm for the management of patients suffering from skin burns caused by radiotherapy (*radiation dermatitis*) or other external events.

## 2. Results & discussion

**Fig. 1:**
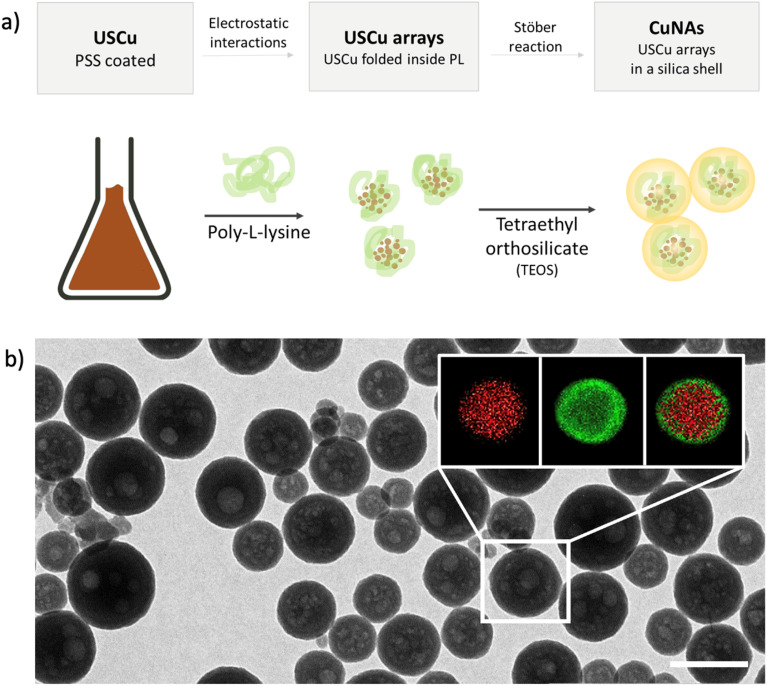
(a) Simplified scheme of synthesis of CuNAs:USCu are prepared, grouped in nano arrays with PL, and subsequently covered with a layer of silica. (b) Transmission electron microscopy image of CuNAs. Scalebar: 200 nm. Energy-dispersive X-ray (EDX) spectroscopy map (inset) confirms the structure of CuNAs, where copper is enclosed in the core of the nano-architecture and Si is present in the shell (Cu in red and Si in green).

Copper nano-architectures (CuNAs) comprise a silica shell (thickness: 12 ± 2 nm) containing ultra-small copper nanoparticles (USCu, <2 nm in diameter) enclosed in an electrostatically folded net of polymers. CuNAs are the most recent member of the wide family of NAs, a class of ultrasmall-in-nano materials implemented for several oncological and antimicrobial applications.^25,30–32^ A schematic explanation of the steps involved in the standardized synthesis that ensures optimal batch-by-batch homogeneity is illustrated in Fig. 1a. A colloidal solution of USCu is prepared that reduces glutathione-complexed copper ions. USCu are then coated with poly(sodium-4-styrene sulfonate) (PSS) and wrapped inside a poly-l-lysine (PL) matrix to create USCu arrays. The arrays are then encapsulated by a hollow silica nano-sphere, resulting in nano-architectures of 155 ± 46 nm in diameter (metal loading 1.1% of total weight) (Fig. 1b and S1 and S2†). Gold nano-architectures (AuNAs) are prepared based on a previously reported standardized protocol and used as a control in the *in vivo* experiment.^24,33^ AuNAs are selected as a suitable control to highlight the effect of copper-based treatment because the only difference between AuNAs and CuNAs depends on the metal contained in the nano-architectures (*i.e.*, gold instead of copper). Hence, the different efficacies of topical treatments can be fully attributed to the action of copper.

CuNAs are constituted by FDA-approved components and are entirely biodegradable. The silica shell is eroded in the cellular environment in 24–48 hours.^27^ Compared to gold NAs, CuNAs demonstrated a slow leakage of copper ions in the physiological buffer even before the complete biodegradation of the silica shell (Fig. S3 and S4†). This effect is due to the chemical nature of copper nanoparticles. Indeed, the surface oxidation of the metal causes the release of copper ions.^20^ This aspect is confirmed by the results of the release experiments performed in HEPES buffer (Fig. S3 and S4†), demonstrating an 8% w/w release of the total initial metal content in 24 h. Thus, CuNAs may release most of their content once inside cells.^27^

Based on previously reported studies, copper ions can act as a booster for wound healing and skin regeneration processes.^20^ These effects depend on the concentration of copper ions. Roughly, if the concentration of copper ions in a cell is slightly increased compared to physiological levels (about 10^−13^ M), a cascade effect causes an accelerated metabolism of the cell (*i.e.*, accelerated wound healing). Copper is involved in the regulation of angiogenin, vascular endothelial growth factor (VEGF), and nerve growth factor (NGF).^20^ If the concentration of copper ions increases further, the metal may be toxic to the cell, generating reactive oxygen species and binding to enzymes and proteins.^20,34,35^ Hence, evaluating *in vitro* the ideal copper concentration to stimulate a therapeutic action without significant toxicity before *in vivo* assessments is pivotal.

**Fig. 2:**
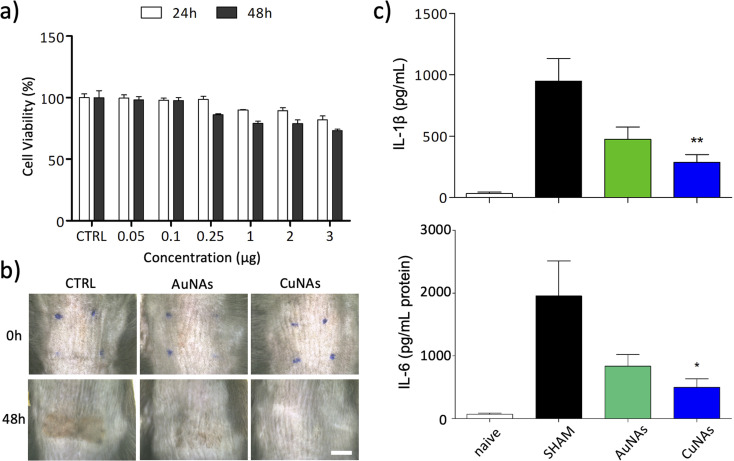
*In vitro* biocompatibility and *in vivo* efficacy of CuNAs for the treatment of skin burns. (a) Viability of human keratinocytes (HaCaT) cells after 24 h and 48 h exposure to various concentrations of CuNAs (μg refers to copper content). Values were normalized to control (no treatment). The average percentage values ± S.D. of three independent experiments are reported. (b) Representative photographs of the skin UVB-exposed mice: untreated (CTRL) and treated with AuNAs or CuNAs. Scale bar: 5 mm. (c) Effects of UVB-induced inflammation on cytokine expression: IL-6 and IL-1β after 48 h of treatment. Results are presented as the average ± SD (*n* = 4 for each group). The data are analyzed using one-way ANOVA, followed by Bonferroni's *post hoc* test, *p < 0.05; **p < 0.01. Naïve: no-irradiated nor treated models. SHAM: irradiated not-treated models.

The effects of CuNAs and AuNAs on the viability of keratinocytes are reported in Fig. 2a and S5,† respectively. The treatments do not induce severe toxicity after 24 h and 48 h at any concentration of tested metals. A moderate 20% reduction in the viability of skin cells is observed after 48 h from the treatment with CuNAs (3 μg in copper). The morphological analysis of the cells confirms the absence of toxicity, and no alterations in the cell shape are noticed either at 24 h or 48 h. Thus, by also considering the reduced integrity of burnt skin, an amount of 50 μg copper/1.5 cm^2^ (Note 1 ESI†) was employed in the *in vivo* evaluations. To apply CuNAs on damaged skin, we prepared a home-made cosmetic cream containing commercial components that are not associated with allergic reactions and are safe for human application.^36^ The cream is prepared by emulsifying a hydrogel with an oil phase and contains the following ingredients: (i) glycerin and betaine (to maintain the moisture and hydration of the skin after application of the cream), (ii) oils and butter (for their emollient properties), (iii) salicylic acid (for the keratolytic and mild anti-inflammatory properties),^37^ (iv) hydrolyzed collagen (stimulates the growth of fibroblasts and the production of new collagen in the dermis),^38^ (v) retinyl palmitate (vitamin A),^39,40^ (vi) *Hamamelis virginiana* water (as a source of tannins).^41^ The home-made cream is finally mixed with CuNAs or, as control, AuNAs (80 mg cream and 50 μg in metal of nano-architectures).

Burns are induced on the skin of immunocompetent mice by 20 min of exposure to ultraviolet B rays (UVB) at 1000 mJ. The exposure of the animal skin to UVB irradiation produces evident erythema with signs of mild burn that led to visible skin lesions and scar formation in the range of 48–96 hours. None of the animals developed blisters. Typical cutaneous manifestations on the skin after UVB exposure are erythema and redness. This pathological condition is also associated with an increased production of inflammatory mediators because UVB irradiation alters the immune function and migration of Langerhans cells and dermal dendritic cells, which produce high levels of cytokines, such as interleukin-1β (IL-1β) and interleukin 6 (IL-6).^42^ After UVB irradiation, an acute inflammation was evident, characterized by the infiltration of neutrophils, and epithelial hyperplasia determined the thickening of the non-keratinized layers of the epidermis. Owing to the inflammatory process, an increase in the number of derma cells was detected. Based on these observations, mild UVB-induced burn in animals affected both epidermis and dermis and could be compared to second-degree damage sunburn in human.^43^ Both AuNAs and CuNAs creams are applied over the irradiated mouse skin, and the cutaneous level of pro-inflammatory cytokines (IL-6 and IL-1β) was assessed at 48 h post UVB irradiation, corresponding to the peak effect of cytokine expression in this model.^44^

The effect of the CuNAs-cream was evaluated and compared on three groups of mice. The reference group (SHAM) did not receive the treatment to evaluate spontaneous recovery from the skin burn without any medication. The second and third groups of mice were treated with cream containing AuNAs or CuNAs, respectively.

Both treatments with AuNAs- and CuNAs-creams enhance skin recovery after one application (Fig. 2b and S6†). Notably, CuNA-cream significantly accelerates recovery by limiting the presence of scars. Moreover, 48 hours after the treatment, CuNAs-cream permitted a full skin recovery with a totally regenerated epidermis. These results are further corroborated by cytokine analysis. One application of CuNAs-cream significantly reduces the expressions of IL-6 and IL-1β to 2 (*p* < 0.05) and 2.3 (*p* < 0.01) times (Fig. 2c). However, the cytokine reduction observed in the animals treated with AuNAs-cream showed no significant difference compared to the untreated group.

**Fig. 3:**
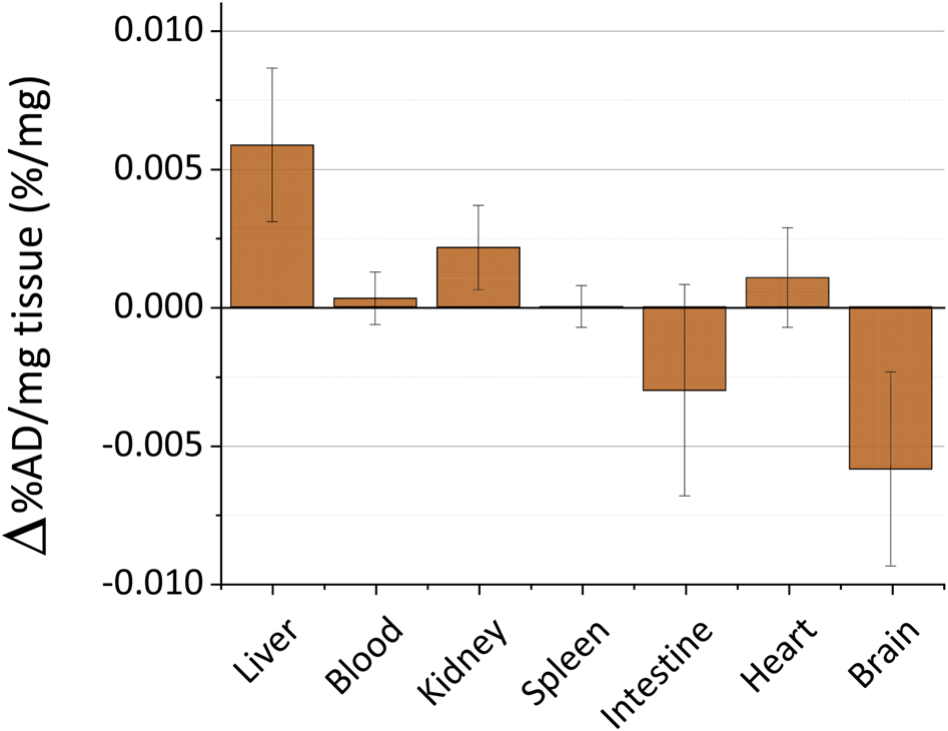
ICP-MS quantification of copper in the organs of treated mice with respect to untreated mice. Copper and relative standard deviations are reported as the percentage of the administered dose normalized by the organ/tissue weight. The copper amount in each organ/tissue (*n* = 3) is subtracted by the naturally occurring copper in the untreated mice. Organs/tissues are collected at 48 h after the treatment.

The toxicity and persistence of metal nanoparticles are topics of pivotal interest to support their translation to clinical practice.^45^ The processes behind metal nanoparticle biodistribution after skin contact are still poorly described, especially on damaged skin.^11^ In our previous studies, we explored the biokinetics of ultrasmall-in-nano silver and gold nano-architectures, demonstrating that the administration pathway drastically determines the fate of the metals.^24,25,46^ Thus, we evaluated the biokinetics of copper by Inductively Coupled Plasma-Mass Spectrometry (ICP-MS) for 48 hours after the skin treatment with the cream containing CuNAs by focusing on target organs. Copper nanoparticles quickly dissolve in the physiological environment by generating copper ions that are easily distributed in organisms and metabolized. No significant trace of copper (Fig. 3) is detected in the blood, kidney, spleen, intestine, or heart. A slight alteration of the copper content compared to the control mice (0.006% of the administered dose) was detected in the liver. It is noteworthy that small oscillations of copper in body organs can result from normal metabolism because copper is involved in many physiological processes.^47^

Copper accumulation in the brain is considered one of the factors that could promote Alzheimer disease.^48,49^ Consequently, we analyzed the copper content in the brain of the treated mice (Fig. 3). Our data exclude copper accumulation in the brain after treatment. This finding is of special interest because the application of nanoparticles on damaged skin can enhance the permeability and spread of the nanomaterials inside the body.^50^ Overall, these results together with the absence of weight loss of the models during the experimental timeframe (Fig. S7†) suggest that CuNAs do not persist or alter the copper metabolism in target organs at a therapeutic amount for burnt skin treatment.

## 3. Conclusions

We demonstrated that rationally designed copper nano-architectures ensure accelerated burnt skin recovery in one application. The reduction in cytokine expression highlights that copper also exerts a significant anti-inflammatory action without inducing significant toxicity. The non-modified levels of copper in organs and blood suggest that the treatment does not considerably alter the systemic metabolism. Notably, this study demonstrates the potential of copper nanoarchitectures in the treatment of burn injuries and opens new horizons in dermatology. The design of CuNAs can be further implemented or modified for applications both in cosmetic and dermatology owing to the versatility of the nano-architectures. It is noteworthy that the treatment is limited to a single dosage in this study although adequate for the assessment of the efficacy and the potential toxicity. However, multiple applications and long-term toxicity will be further evaluated. Moreover, further studies will be directed towards the exploitation of the efficient regeneration abilities of CuNAs for the management of *radiation dermatitis* and, in general, severe skin burns.

## 4. Materials and methods

### 4.1 Reagents

Reagents for nano-architecture synthesis were purchased from Sigma-Aldrich, unless specified otherwise, and were used without further purification. *Butyrospermum parkii* butter (shea butter), caprylic/capric triglycerides (TEGOSOFT® CT), POLYSORBATE 20 (Tween 20®), methyl glucose sesquistearate (TEGO® Care PS), *Passiflora edulis* seed oil (maracujà oil), *Macadamia ternifolia* seed oil (macadamia oil), betaine, glycerin, algin (sodium alginate), titanium dioxide, salycilic acid, *Jasminum officinale* flower extract (jasmine essential oil), *Hamamelis virginiana* water, sodium benzoate, potassium sorbate, xanthan gum, citric acid, hydrolyzed collagen, lemongrass essential oil, retinyl palmitate (vitamin A palmitate), tocopherol (vitamin E), and Mica were purchased from ZenStore.it.

### 4.2 Synthesis of nano-architectures

For the synthesis and characterization of AuNAs (TEM, metal loading), refer to the include references.^24,25,33^

#### 4.2.1 Synthesis of copper seeds

To 20 mL of milliQ® water, 200 μL of reduced glutathione solution 100 mM (30.72 mg mL^−1^) and 200 μL of CuSO_4_ aqueous solution 25 mM (3.99 mg mL^−1^) are added. After the solution becomes milky, 200 μL of sodium borohydride (NaBH_4_) solution 8 mg mL^−1^ are quickly added. The mixture is vigorously stirred for 2 minutes assuming a pale-yellow color. Then, 10 μL of poly(sodium-4-styrene sulfonate) (PSS) 70 kDa (30% aqueous solution) are added, and the reaction is stirred at room temperature for 10 minutes.

#### 4.2.2 Synthesis of CuNP arrays

75 μL of 40 mg mL^−1^ poly-l-lysine 15–30 kDa (PL) are added under gentle stirring for 20 minutes at room temperature. The arrays are collected by 3 minutes of centrifugation at 13 400 rpm and redispersed in 4 mL of milliQ® water sonicating for 1 minute.

#### 4.2.3 Synthesis of CuNAs

The arrays are used as templates for silica shell formation through a modified Stöber reaction. In a 100 mL round-bottomed flask, 70 mL of ethanol are aged for 5 minutes with 42 μL of tetraethyl orthosilicate (TEOS, 98%). 2 mL of CuNP arrays are added. After 5 minutes, 141 μL of dimethylamine (DMA) (40% stock, 428 mg mL^−1^) are further added. The resulting solution is stirred for 3.5 hours at room temperature. CuNAs are collected by 30 minute centrifugation at 4000 rpm and washed twice with ethanol to remove unreacted precursors. The bigger CuNAs are eliminated by performing a short spin (14 seconds at 14 000 rpm), and the recovered supernatant is washed once more in ethanol and stored in 1 mL of ethanol at −20 °C.

### 4.3 Characterization of nano-architectures

#### 4.3.1 Dynamic light scattering (DLS) measurements

The hydrodynamic diameter and the zeta potential measurements are acquired using a Malvern Zetasizer Nano ZS90 (Malvern Panalytical Ltd, Malvern, UK). Before the measurements, CuNAs are resuspended in 1× phosphate-buffered saline (PBS) at pH 7.4 and sonicated for 2 minutes. The values are reported as the average of three consecutive measurements.

#### 4.3.2 Electron microscopy

Transmission electron microscopy (TEM) images of nanoparticles are collected using a ZEISS Libra 120 TEM (Carl Zeiss NTS, Oberkochen, Germany), operating at an accelerating voltage of 120 kV and equipped with an in-column omega filter. The CuNA suspension is dropped on 300-mesh carbon-coated copper grids and dried before observation.

#### 4.3.3 Energy-dispersive X-ray (EDX) spectroscopy analysis

EDX spectroscopy is performed with a JEOL JEM-F2000 Multi-purpose, working at 200 kV and equipped with a Schottky-FEG source and high-counting SDD EDX detector. EDX maps are performed in scanning mode, with a probe size of 1 and a counting time of 2 minutes.

#### 4.3.4 Inductively coupled plasma-mass spectrometry (ICP-MS) analysis

Copper quantification is performed by diluting 10 μL of CuNA suspension in 200 μL of nitric acid 65% Suprapur®. The sample is digested at 200 °C under microwave irradiation with a CEM Discover SP-D digestion microwave (CEM, Matthews, NC, USA). The resulting solution is diluted in 3 mL of 3% nitric acid solution, and the Cu content is assessed by ICP-MS Agilent 7700 (Agilent Technologies, Santa Clara, CA, USA) analysis against a standard calibration curve with 10 ppm Hg in nitric acid solution as the internal standard.

#### 4.3.5 *In vitro* copper release studies

The *in vitro* copper release studies are carried out using a dialysis membrane with molecular weight cut-off (MWCO) 10 kDa. CuNAs are resuspended in 100 μL HEPES buffer 20 mM and sealed in a dialysis membrane kept in 15 mL HEPES buffer 20 mM at 37 °C. At the defined time points, 3 mL of samples are withdrawn and substituted with 3 mL of fresh buffer for up to 7 days. The collected samples are analyzed for copper quantification by ICP-MS Agilent 7700 (Agilent Technologies, Santa Clara, CA, USA) against a standard calibration curve with 10 ppm Hg in nitric acid solution as the internal standard.

### 4.4 CuNA-cream preparation

#### 4.4.1 Formulation of the gel cream

A gel oil-in-water (O/W) cream is prepared by mixing a hydrogel and an oil phase. For 100 g of O/A cream, the water phase is prepared as follows. 0.2 g of citric acid is dissolved in 90 mL of MilliQ® water. Potassium sorbate (0.25 g), sodium benzoate (0.25 g), *Hamamelis virginiana* water (0.3 mL) and collagen (0.2 mL) are dissolved in the solution. The pH is around 5. Then, 1.88 g of betaine is added to the mixture. Xanthan gum (0.23 g) is moistened using 1.88 g of glycerin and then added to the water solution. Sodium alginate (1 g) and the mica powders are first mixed and then added to the water solution. The resulting mixture is vigorously mixed using a pestle to obtain the hydrogel.

The oil phase is prepared as follows. Shea butter (5.8 g) is bain-marie heated until complete fusion. Polysorbate 20 (3 g) and methyl glucose sesquistearate (3 g) are dissolved in the fused shea butter. When the latter is completely dissolved, caprylic/capric triglycerides (4 g), *Passiflora edulis* seed oil (2.5 g), *Macadamia ternifolia* seed oil (2.5 g), retinyl palmitate (0.05 g) and tocopherol (0.05 g) are added. Salicylic acid (0.5 g) and titanium dioxide (1 g) are mixed and ground before being suspended in the oily mixture. Lemongrass and jasmine essential oils (0.1 g and 0.5 g, respectively) are added to the oil phase immediately before emulsifying the two phases. The oil phase was dropped onto the hydrogel under vigorous stirring. The emulsion was stirred until it turned white and homogeneous.

#### 4.4.2 Preparation of CuNA-cream

CuNAs (50 μg Cu) are mixed with 80 mg cream and topically applied to the injury on the mouse.

### 4.5 Viability experiments

Human keratinocyte cell lines (HaCaT, Cell Line Service, Heidelberg, Germany) are cultured in DMEM with 10% fetal bovine serum (FBS) and 2 mmol L^−1^l-glutamine at 37 °C in an atmosphere of 5% CO_2_ and 95% air. The *in vitro* cytotoxicity is determined with CellTiter-Glo® Luminescent viability assay (Promega, MI, Italy) measuring ATP levels and reported as the percentage survived relative to control cells. Cells are incubated in 96-well plates until confluence is reached (initial density: 3.5 × 10^5^ in 100 μL). After 24 h, cells are rapidly rinsed with pre-warmed PBS with Ca^2+^/Mg^2+^, and the extraction medium is replaced with the NAs-medium (control samples are treated with medium processed as the extractions), and cells are incubated for an additional 24 h and 48 h. NAs-mediums are prepared by mixing NAs in cell medium at various concentrations. According to ISO10993-5 guidelines, as the cell viability, normalized over control, of the sample extracts is higher than 70%, all materials are considered biocompatible. Data represent the mean ± SD of three independent experiments. The impact of CuNAs on cell morphology is also monitored using the LEICA DMI6000B inverted microscope.

### 4.6 *In vivo* studies on UVB-induced skin inflammation

Animal experiments are conducted following the European Communities Council Directive (Directive 2010/63/EU of 22 September 2010) and approved by the National Council on Animal Care of the Italian Ministry of Health (746/2019-PR). Ventilated cages are employed with free access to food, water, humidity (50 ± 10%), temperature (21 ± 2 °C), and light (10 and 14 hours of light and dark, respectively). Based on the “3Rs concept”, the number of animals used in this study and their potential suffering is reduced.

UVB inflammation was induced in 8 week-old male C57BL/6J mice (Charles River, Calco, Italy) after anesthesia (a mixture of ketamine (10%) and xylazine (5%), intraperitoneal injection). The burn wounds are induced as reported in the literature after the animal dorsal skin is shaved with an electric clipper.^44^ The burnt area of the murine models corresponds to about 3.7% of the total body area (corresponding to about 555 cm^2^ for a human body). Following burn induction, the exposed area is immediately treated by placing CuNAs or, as a control, AuNAs (80 mg cream and 50 μg in metal of nano-architectures) applied once after the UV exposure covered with Tegaderm™ to prevent the mice from removing the treatment. Naïve mice are treated with the same procedures without being irradiated and without any pharmacological treatment. After 48 h from the UVB burn induction, animals are sacrificed and samples from UVB-exposed and non-exposed skins are stored at −80 °C. Subsequently, each sample is homogenized and centrifuged, and the supernatant is saved at −80 C°. The cytokine expression is measured using an ELISA quantikine kit (R&D system) based on the manufacturer's instructions. For each sample, the cytokine concentration is normalized against the total protein content, as measured using the bicinchoninic acid (BCA) assay (Thermo Scientific, Rockford, IL, USA).

### 4.7 Statistical analysis

One-way ANOVA is utilized to evaluate statistical significance, followed by Bonferroni's *post hoc* test. GraphPad Prism 5 is utilized for all statistical analysis (GraphPad Software Inc. San Diego, CA, USA). Results with a *p*-value < 0.05 are considered statistically significant.

For metal quantification *via* ICP-MS, each organ sample is measured 3 times and the software of the Agilent 7700 automatically compares the average value to a freshly measured calibration curve. Values of the same organ in different mice (at least 3 mice) are then compared, and the average value is considered with the relative standard deviation calculated with Microsoft Excel.

## Conflicts of interest

The authors declare no conflict of interests.

## Supplementary Material

NA-005-D2NA00786J-s001
