# Supplementary material for: Copper nano-architecture topical cream for the accelerated recovery of burnt skin
Source: Nanoscale Adv. 2023 Jan 18;5(4):1212–9. doi: 10.1039/d2na00786j (PMC9926901; doi:10.1039/d2na00786j)
Supplement: NA-005-D2NA00786J-s001 [file NA-005-D2NA00786J-s001.pdf]

Electronic Supplementary Material (ESI) for Nanoscale Advances.
This journal is © The Royal Society of Chemistry 2023

Copper nano-architectures topical cream for the accelerated recovery of burnt skin

Maria Laura Ermini,[1,§] Maria Summa,[2,§] Agata Zamborlin,[1,3] Valentina Frusca,[1] Ana Katrina Mapanao,[1] Enrico Mugnaioli,[4] Rosalia Bertorelli,[2,*] and Valerio Voliani[1,5*]

[1] Center for Nanotechnology Innovation@ NEST, Istituto Italiano di Tecnologia, Piazza San Silvestro, 12 – 56127, Pisa, Italy

[2] Translational Pharmacology, Istituto Italiano di Tecnologia, Via Morego, 30 – 16163, Genoa, Italy

[3] NEST-Scuola Normale Superiore, Piazza San Silvestro, 12 - 56127, Pisa, Italy

[4] Department of Earth Sciences, University of Pisa, Via S. Maria 53, 56126 Pisa, Italy

[5] Department of Pharmacy, University of Genoa, Viale Cembrano, 4 - 16148, Genoa, Italy

§ These Authors contributed equally

* Corresponding Authors: rosalia.bertorelli@iit.it, valerio.voliani@unige.it

Figure S1

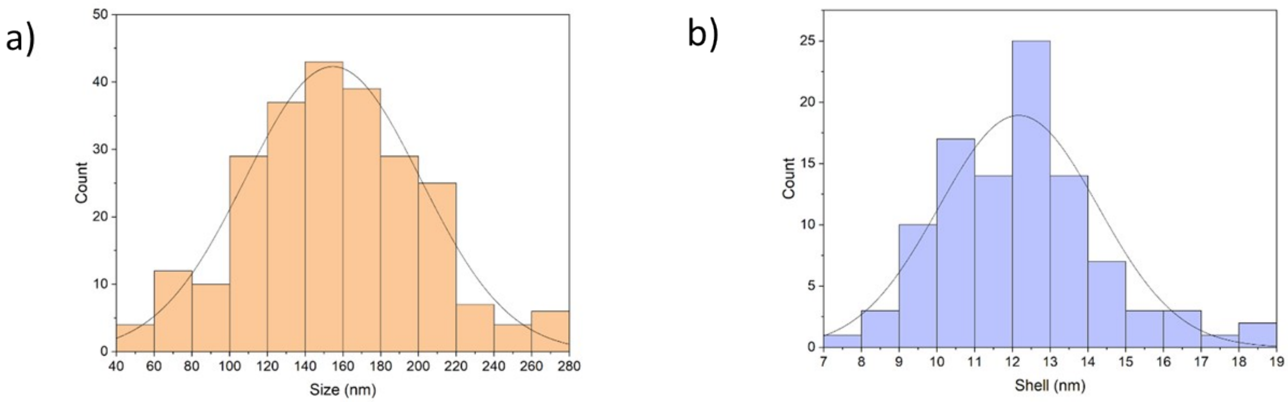

Figure S1. CuNAs diameter dispersion (155±46 nm) (a), and silica shell thickness (12±2 nm) (b).

Figure S2

	in EtOH	in PBS
Size (nm)	125±25<br>PDI:<br>0.677±0.559	403±10<br>PDI:<br>0.079±0.054
ζ Potential (mV)	-35.4±0.8	-5.88±1.5

Figure S2. CuNAs hydrodynamic diameter and ζ-potential in ethanol and PBS. Data differences are associated to the different medium.

Figure S3

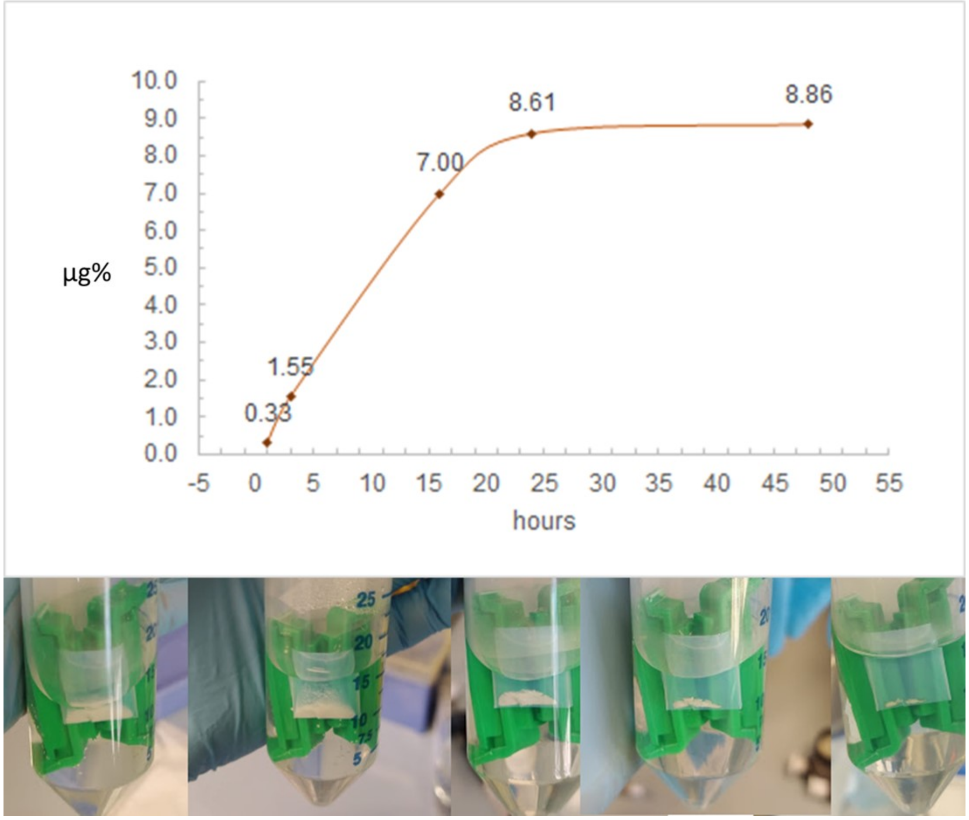

Figure S3: Amount of copper (reported as % µg) released from CuNAs *vs* time (upper panel). Images of the dialysis experiment at different time points (bottom).

Figure S4

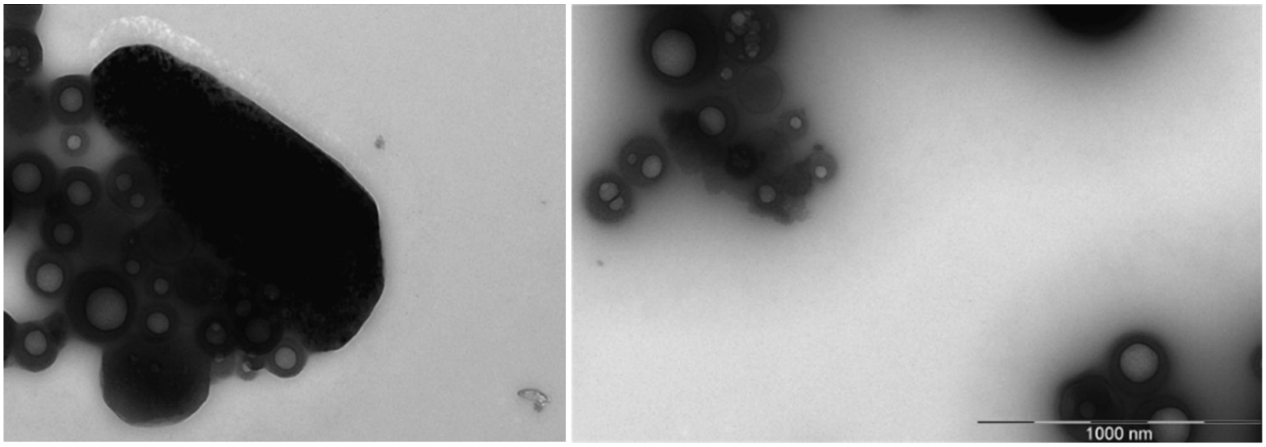

Figure S4. TEM images of CuNAs after release experiments after 50 hours. The shell is evidently eroded, and nanoparticles are still present in deformed arrays.

Figure S5

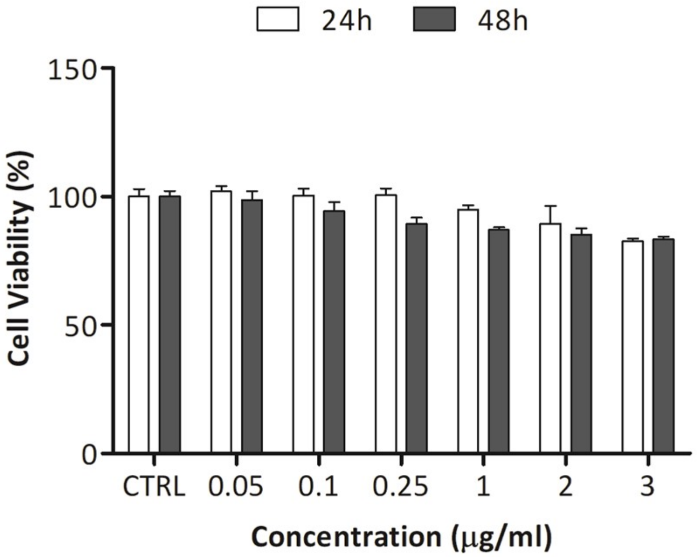

Figure S5. Viability of HaCaT cells 24h and 48h after exposure to different concentrations of AuNAs (μg refers to gold content).

Figure S6

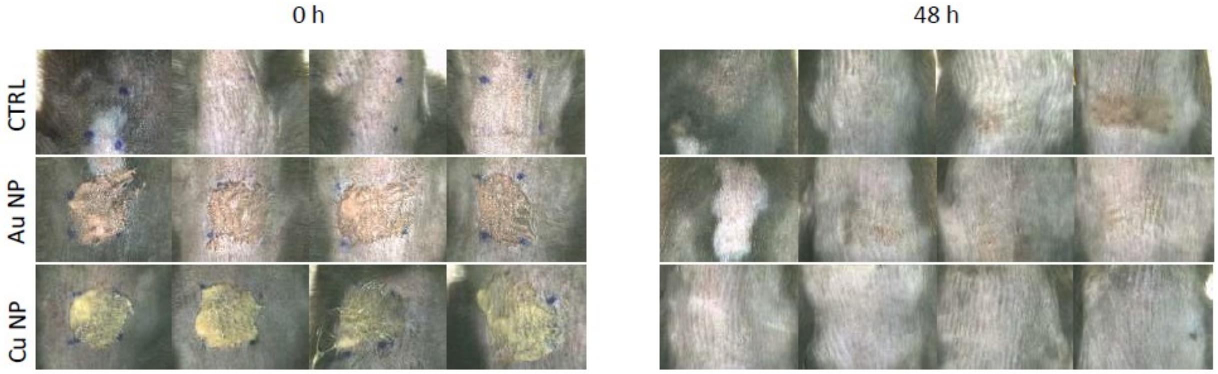

Figure S6. Representative photographs of the skin of UVB exposed mice: untreated (CTRL) and treated with AuNAs or CuNAs.

Note 1

Knowing the maximum tested copper amount in cell culture evaluation (10000 cells seeded), we calculate that each cell is roughly treated with 0.3 ng of copper. Thus, we estimate that the maximum safe amount of copper to employ on 1.5 cm^2 burnt skin area (about 9M of cells) corresponds to 2.7 mg of copper on mice.

Figure S7

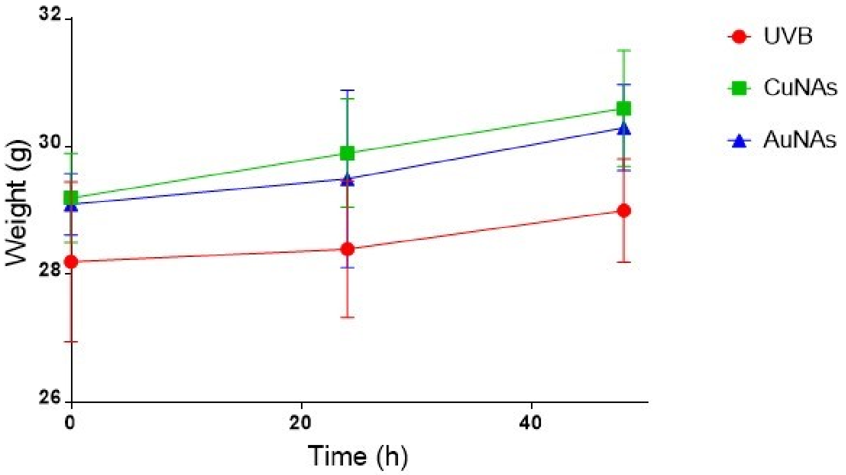

Figure S7. Body weight variation of the animal models during the experimental timeframe. No significant changes were observed.
